# Understanding Early-Life Adaptive Immunity to Guide Interventions for Pediatric Health

**DOI:** 10.3389/fimmu.2020.595297

**Published:** 2021-01-21

**Authors:** Eleanor C. Semmes, Jui-Lin Chen, Ria Goswami, Trevor D. Burt, Sallie R. Permar, Genevieve G. Fouda

**Affiliations:** ^1^Duke Human Vaccine Institute, Duke University, Durham, NC, United States; ^2^Medical Scientist Training Program, Duke University, Durham, NC, United States; ^3^Children’s Health and Discovery Initiative, Department of Pediatrics, Duke University, Durham, NC, United States; ^4^Division of Neonatology, Department of Pediatrics, Duke University, Durham, NC, United States

**Keywords:** infant immunity, adaptive immunity, pediatric infectious diseases, neonatal T cells, vaccines, neonatal B cells, adjuvants, maternal antibody transfer

## Abstract

Infants are capable of mounting adaptive immune responses, but their ability to develop long-lasting immunity is limited. Understanding the particularities of the neonatal adaptive immune system is therefore critical to guide the design of immune-based interventions, including vaccines, in early life. In this review, we present a thorough summary of T cell, B cell, and humoral immunity in early life and discuss infant adaptive immune responses to pathogens and vaccines. We focus on the differences between T and B cell responses in early life and adulthood, which hinder the generation of long-lasting adaptive immune responses in infancy. We discuss how knowledge of early life adaptive immunity can be applied when developing vaccine strategies for this unique period of immune development. In particular, we emphasize the use of novel vaccine adjuvants and optimization of infant vaccine schedules. We also propose integrating maternal and infant immunization strategies to ensure optimal neonatal protection through passive maternal antibody transfer while avoiding hindering infant vaccine responses. Our review highlights that the infant adaptive immune system is functionally distinct and uniquely regulated compared to later life and that these particularities should be considered when designing interventions to promote pediatric health.

## Introduction

Despite tremendous progress in recent decades, infectious diseases remain a leading cause of morbidity and mortality in pediatric populations worldwide (1–3). Infectious diseases cause nearly 25% of deaths in the neonatal period (from birth to one month of age) and up to one third of deaths in children under 5 years of age (1–3). Vaccines are one of the most cost-effective interventions to address the global burden of pediatric infectious diseases, and the implementation of early life immunizations has reduced deaths in neonates and children across the world (4). Due to differences in the early life and adult immune systems, it is increasingly appreciated that an in-depth understanding of early life immunity is crucial for the development of effective pediatric vaccines and for optimizing pediatric vaccine schedules.

While recent reviews have outlined differences between the infant and adult immune system, they focus on innate immunity given its key role in defense in early life [reviewed in (5–7)]. Because vaccine-induced protection largely depends on acquired immunity, we have instead focused this review on early life adaptive immunity. Herein, we present a detailed overview of T and B cell development as well as the unique factors regulating early life adaptive immunity. We review T cell and humoral responses to pathogens and vaccines in early life, which reveal that adaptive immune responses can be generated in infancy but that these are generally attenuated compared to later in life. We also discuss how passive maternal antibody transfer impacts early life adaptive immunity and may be harnessed to protect neonates. Our review focuses on human studies of early life adaptive immunity, yet we have also integrated evidence from animal models where appropriate (see **Box 1**). Our goal is that this review can inform the rational design of vaccines and other immune-based interventions to combat pediatric infectious diseases. Understanding early life immunity is particularly critical for vaccine development since vaccines and adjuvants traditionally have not been tailored to engage the neonatal or infant adaptive immune response. Therefore, applying knowledge of early life adaptive immunity in vaccine development can substantially improve the efficacy and impact of pediatric vaccines.

Box 1 **Key considerations for studies of early life immunity. Many studies of early life immunity use neonatal mice or human umbilical cord blood, yet these model systems have important caveats: 1) murine and human immune cell ontogeny differ substantially, especially regarding T cell development [(8, 9) reviewed in (10–12)], 2) immune responses measured in umbilical cord blood are distinct from those detected during prenatal and postnatal life (13), and 3) the milieu of cytokines, corticosteroids, and metabolites generated during labor can significantly modulate immune responses detected in cord blood [reviewed in (14, 15)]. While the use of model systems is critical to complement human studies, it is important to consider possible limitations when interpreting different studies of early life immunity.**

## T Cells in Early Life

### T Cell Development and the Neonatal T Cell Compartment

#### Early Life T Cell Development and Immunophenotypes

**Figure 1:**
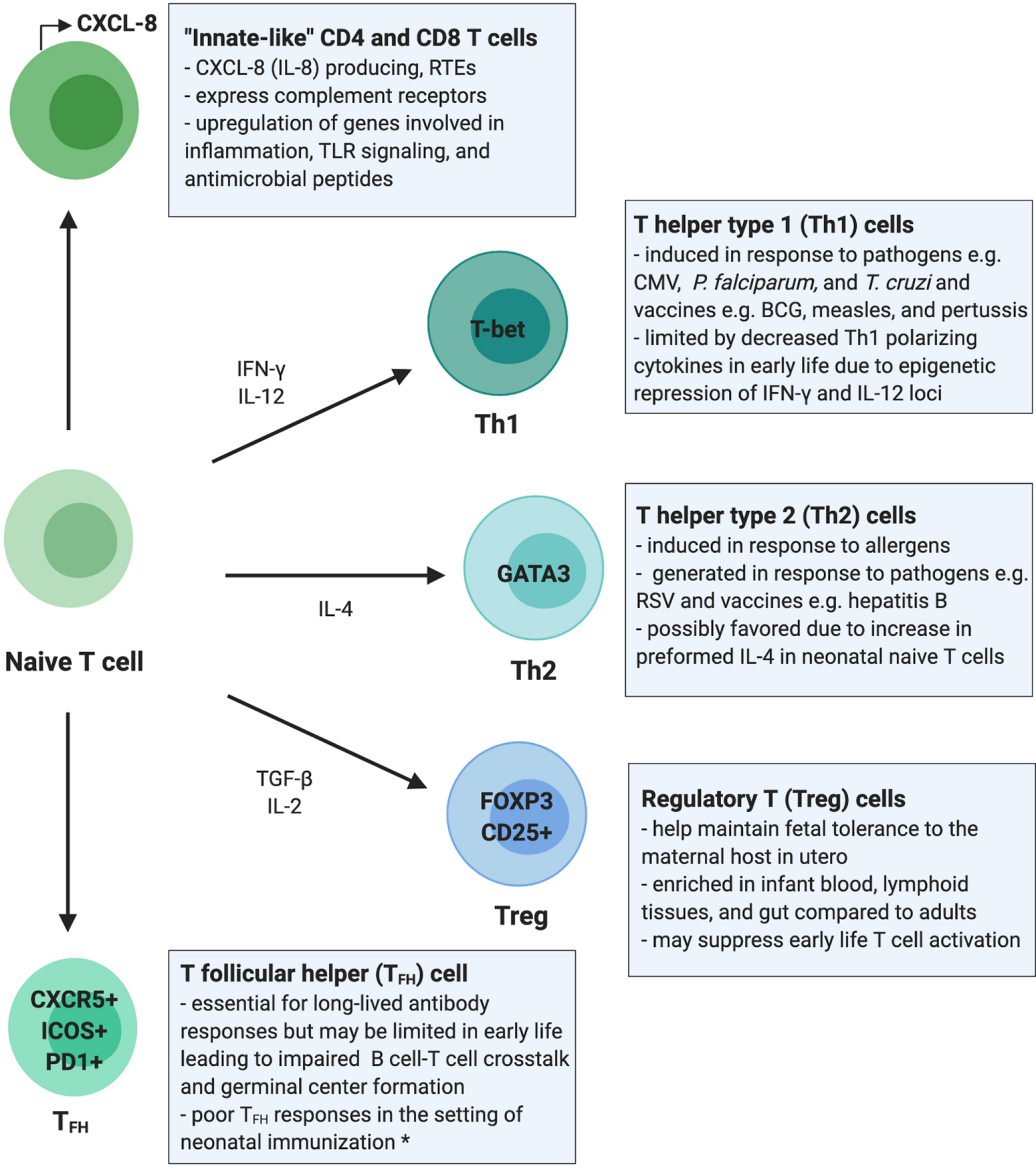
Summary of the T cell compartment in early life. This overview of the infant T cell compartment summarizes evidence from human studies unless otherwise stated. Infants rely on “innate-like” CD4+ and CD8+ T cells, which are more likely to be RTEs and to signal through innate immune pathways such as complement receptor and TLR signaling, promote inflammation, and respond with antimicrobial peptides rather than classic antigen-specific adaptive immune responses. T helper type 1 (Th1) cells are generated in response to certain pathogens and vaccines in early life; however, epigenetic regulation of cytokine loci may limit these responses. T helper type 2 (Th2) cells are generated in response to allergens and common environmental antigens encountered in early life and only rarely in response to pathogens or vaccines. Regulatory T cells (Tregs) maintain fetal immunotolerance in utero and are present in higher numbers and proportions in early life blood and peripheral tissues, which may aid in promoting tolerance to microbial colonization but may impair mucosal and systemic T cell activation and adaptive immune responses. T follicular helper cell (T_FH_) responses are impaired in neonatal murine models but have not been studied extensively in human neonates. The restricted function of T_FH_ cells in early life may be due to immaturity in co-activation signals required for B and T cell crosstalk and may impair generation of germinal center responses. *data shown in mice. RTEs, recent thymic emigrants; CMV, cytomegalovirus; RSV, respiratory syncytial virus; *P. falciparum*, *plasmodium falciparum*; *T. cruzi*, *Trypanosoma cruzi*.

The T cell compartment in early life is uniquely positioned to respond to diverse immunological demands, such as balancing immunotolerance in utero and during microbial colonization with defense against pathogens (12). These divergent demands result in highly stimulus dependent T cell responses in early life (**Figure 1**). Human T cell development begins in utero, as the thymus starts producing T cells at 12–14 weeks of gestation, and the neonatal T cell receptor (TCR) repertoire is diversified by 26 weeks of gestation (16–19). Distinct waves of human T cell development occur in utero, yet the T cell compartment continues to evolve dynamically after birth. In contrast to other immune cell compartments (*e.g.*, B, natural killer, and dendritic cells) that converge with adult immunophenotypes by 3 months of age, infant and adult T cell compartments remain phenotypically distinct at least for the first 2 years of life (13, 20).

Infants have high inter-individual variability in CD4+ and CD8+ subsets [*i.e.*, proportion of naïve, central memory (T_CM_), effector memory (T_EM_), and tissue-resident effector memory T cells (T_EMRA_)], yet overall the neonatal T cell compartment is dominated by naïve T cells (13). The majority of these naïve T cells are recent thymic emigrants (RTEs), which harbor unique effector functions (18, 21–23). RTEs are biased towards innate-like immune signaling and preferentially differentiate into induced regulatory T cells (iTregs), which limits their role in adaptive T cell responses (22, 24). Despite the predominance of naïve T cells, T_EM_ are present in cord blood even in the absence of intrauterine infection, suggesting that T cell memory is generated during normal fetal development (25, 26).

T cells from pediatric organ donors (2 months to 2 years of age) are also more likely to be naïve and RTEs compared to T cells from adults (20). This increased proportion of naïve T cells and RTEs has been observed across T cell compartments in blood, lymphoid tissue, lung, and the intestine (20). In general, infants have fewer T_EM_ cells compared to adults except in the lung and small intestine where proportions are comparable (20). Given the high burden of respiratory and diarrheal diseases in infants, it is interesting to note that effector T cell memory may be relatively enhanced in the lung and small intestine in early life compared to other tissues; however, the functional capabilities of these T_EM_ populations are largely unknown. Moreover, the increased ratio of Tregs : T_EM_ cells in these tissues may hinder the ability to mount an effector-memory response (20). Thus, while T_EM_ responses are generated in utero and present in mucosal tissues (*i.e.*, lung and small intestine) in early life, it is unclear how effective they are in mounting adaptive responses. Overall, the relative enrichment of antigenically naïve T cells and RTEs compared to memory and effector T cells likely contributes to blunted adaptive T cell responses in infancy.

#### Innate-Like Functions of T Cells in Early Life

In addition to phenotypic differences, adult and infant T cells have distinct functional responses. Notably, cord and neonatal peripheral blood contain fewer IFN-γ and IL-17A producing but significantly more CXCL-8 producing T cells compared to adult blood (23, 27, 28). CXCL-8 is an innate immune effector, and CXCL-8 producing T cells can co-express complement receptors (CR), which also function in innate immune cell signaling (23). These CXCL-8 producing T cells are enriched in the neonatal T cell compartment, more likely to be RTEs, and may act as innate-like precursors to the classic adaptive proinflammatory IFN-γ producing T cells (22).

Over the first 3 months of life, CXCL-8 plasma concentrations decrease whereas IL-17A concentrations increase, reflecting a shift from innate-like to canonical adaptive cytokine production (13). However, T cells from older children (aged 5–16 years) still show decreased proinflammatory cytokine secretion (*e.g.*, IFN-γ and IL-17A) compared to adults following *ex vivo* stimulation (29). These differences in early life cytokine production highlight the importance of CXCL-8 and suggest that neonatal T cells respond preferentially with innate rather than adaptive responses favored in adulthood (**Figure 1**). This skewing of the early life T cell compartment towards innate-like responses may need to be overcome in order to elicit durable adaptive T cell responses in infancy.

#### Mechanisms Regulating T Cells in Early Life

Cell-intrinsic (*i.e.*, transcriptional, epigenetic) and cell-extrinsic (*i.e.*, cytokines, cell-to-cell communication) mechanisms that regulate T cell responses are distinct in early life compared to adulthood. For instance, fetal and neonatal T cells have a transcriptional landscape that favors tolerogenic and innate-like cytokine production over proinflammatory T cell responses (30). Moreover, when comparing transcriptional pathways in preterm, term, and 3 month old infants, younger neonates exhibit a downregulation of IFN-γ production and T cell proliferation, and upregulation of IL-10 and CXCL-8 biosynthesis (13). These results suggest that neonatal T cells are biased towards innate immune signaling and immunosuppression, as IL-10 promotes Treg differentiation and suppresses T cell activation. Transcriptional differences between naïve CD8+ T cells from cord and adult blood further supports the hypothesis that T cells in early life harbor a gene expression program favoring innate over adaptive T cell functions (*i.e.*, antigen recognition) (31). Naïve CD8+ T cells from cord blood upregulate innate immune genes in toll-like receptor (TLR) signaling, inflammation, and antimicrobial peptides whereas those from adults highly express cell cytotoxicity and TCR-signaling genes (31). Mechanistic work in mouse models further supports the notion that early life T cells employ transcriptional programs to promote rapid innate immune responses over antigen-specific memory responses (32, 33).

Neonatal T cells also have a distinct epigenetic landscape compared to adult T cells, as reflected by differences in DNA methylation, chromatin, histone modifications, and micro RNA (miRNA) expression [reviewed in (12)]. In fetal CD4+ T cells, IL-2 and IFN-γ production is blunted by expression of the transcription factor Helios (34). Moreover, in cord blood, the IFN-γ promoter of naïve CD4+ T cells is heavily methylated, which correlates with lower levels of IFN-γ secretion (35). Chromatin accessibility at other cytokine loci (*e.g.*, IL-12 and IL-13) in neonatal T and dendritic cells differs from adults, suggesting that chromatin remodeling occurs during the transition from neonatal to adult T cell phenotypes (36, 37). Epigenetic differences in chromatin, histone modifications, and miRNA expression in cord versus adult T cells likely drive differences in gene expression and function [(31, 38–40), reviewed in (12)]. Most of these findings are from cord blood and mouse studies, thus additional studies are needed to understand the transcriptional and epigenetic mechanisms governing T cells postnatally. Such work may reveal novel therapeutic targets for modulating infant T cells to promote long-lasting adaptive responses.

#### Immunotolerance and Regulatory T Cells (Tregs) in Early Life

Mounting evidence suggests that a strong bias towards Treg development in early life tempers adaptive immune responses. Fetal immunotolerance in utero is essential and is maintained by immunosuppressive Tregs, which hamper proinflammatory T cell activity and promote tolerance to the maternal host [reviewed in (41, 42)]. Tregs are enriched in fetal lymphoid tissues and they suppress non-Treg T cell proliferation, activation, and IFN-γ production (30, 43). Moreover, expression of Helios in fetal naïve CD4+ T cells contributes to an epigenetic predisposition to Treg differentiation (34). Both fetal, and to a lesser extent, neonatal CD4+ T cells are poised to differentiate into Tregs upon TCR engagement (30, 40, 44).

Treg persistence postnatally and their role in modulating adaptive immune responses in infancy is unclear. During fetal development, the proportion of Tregs declines with gestational age from 15–20% in the second trimester to ~5% of total CD4+ T cells in cord blood, which is comparable to adult blood (44, 45). However, recent work has demonstrated that Tregs are a highly enriched and compartmentalized T cell subset in early postnatal life (20). When comparing T cell compartments from pediatric (2 months to 2 years of age) to adult organ donors, Thome et al. found that Tregs accounted for 10-20% of total CD4+ T cells in pediatric blood, lung, intestine, and lymphoid tissues versus only ~5% in adult donor tissues (20). This predominance of Tregs was directly correlated with age, as highlighted by decreasing proportions of Tregs over time. The importance of Tregs in modulating infant immune responses across diverse sites (*e.g.*, blood, lymphoid, and mucosal tissues) has been underexplored. Tregs likely regulate appropriate local mucosal immune responses during microbial colonization, as Tregs may protect against necrotizing enterocolitis in preterm infants by promoting tolerance during newborn gut microbial colonization (46). Because the immunosuppressive effects of these Tregs may increase infectious disease susceptibility and could dampen vaccine responsiveness in infancy, further research is crucial to gain a deeper understanding of their abundance and function in early life.

#### T Helper (Th) Cell Responses in Early Life

It is generally believed that CD4+ T cells are biased towards T helper type 2 (Th2) differentiation in early life [reviewed in (11, 47, 48)]. This tendency towards Th2 differentiation was first observed in neonatal mice, but the evidence for Th2 bias in human neonates is less clear. Naïve CD4+ T cells can differentiate into Th subsets including Th1, Th2, and Th17 cells, which produce IFN-γ, IL-4/IL-5/IL-13, and IL-17/IL-22, respectively. Th1 cells are proinflammatory and defend against intracellular pathogens (*e.g.*, viruses), whereas Th2 cells protect against parasitic infections and have been implicated in atopic diseases (*e.g.*, allergy and asthma), and Th17 cells protect against extracellular pathogens (**Figure 1**). While neonatal innate immune cells produce more Th2 than Th1-polarizing cytokines (49, 50), Th responses in early life appear to be highly stimulus dependent.

Recently, a population of naïve CD4+ T cells has been identified in cord blood and infant adenoids that highly express IL-4 when stimulated under multiple conditions (*e.g.*, anti-CD3/anti-CD28, PMA/Ionomycin, TGF-β). However, IFN-γ is also expressed by these T cells under some conditions (*e.g.*, PMA/Ionomycin, IL-1β, IL-12), indicating that these cells generate both Th1 and Th2 type responses (51). Additional evidence that the stimuli and cytokine milieu strongly dictate early life Th differentiation is apparent from studies of neonatal T cell responses to allergen. T cells isolated from cord blood produce Th2 type cytokines when stimulated by environmental allergens *ex vivo* (52). Similarly, T cells from infants (<2 years of age) stimulated with allergens favor Th2 over Th1 responses (53). Infants (<3 months of age) infected with RSV also generate high levels of IL-4 and no detectable IFN-γ, suggesting a Th2 biased response (54). RSV also elicits a Th2 type response in neonatal mice, and early life RSV infections in humans have been implicated in the development of atopy and asthma [(55) reviewed in (56)]. Thus, Th2 responses to RSV infection may be pathogen-specific and reflective of a complex interplay with Th2-mediated disease risk. Overall, it is clear that infants mount Th2 responses against allergens and RSV in early life but less apparent whether this represents a global Th2 bias.

Infants (<3 months of age) infected with influenza or parainfluenza virus generate IFN-γ and IL-4/IL-5, suggesting they mount both Th1 and Th2 responses (54). Moreover, a prospective analysis of Th1 and Th2 cytokine levels in newborns followed until 3 months of age did not show Th2 skewing (57). T cells from lymph nodes of pediatric organ donors (<2 years of age) also do not demonstrate a Th2 bias after stimulation (20). Importantly, it is unclear if Th responses studied *ex vivo* recapitulate *in vivo* function. However, both *in vivo* and *ex vivo* evidence suggests that the stimulation (*e.g.*, allergen, pathogen, TCR/co-stimulation, mitogen) and cytokine milieu (*e.g.*, strongly Th-polarizing, proinflammatory, anti-inflammatory) largely dictate Th responses in early life (**Figure 1**).

### T Cell Responses to Pathogens and Vaccines in Early Life

#### Early Life T Cell Responses to Pathogens

Studies of fetal and neonatal immune responses to infections have greatly advanced our understanding of the functional capabilities of T cells in early life [reviewed in (58)]. Fetuses mount pathogen-specific CD8+ and CD4+ T cell responses against human immunodeficiency virus (HIV), cytomegalovirus (CMV), *Trypanosoma cruzi*, and *Plasmodium falciparum* [(59–63) reviewed in (64–66)]. Fetuses exposed to *P. falciparum* and *T. cruzi* generate CD4+ T cell responses that release proinflammatory cytokines when re-stimulated, highlighting that adaptive T cell memory is elicited in utero (62, 67). Moreover, infants exposed to *P. falciparum* in utero produce antigen-specific CD4+ and CD8+ T cell responses that undergo memory differentiation (63). These *P. falciparum* specific CD4+ T cell responses in cord blood correlate with protection against malaria infection in the first 2 years of life, suggesting that priming of pathogen-specific CD4+ T cell responses in utero may confer protection later in life (63).

Infectious exposures can impact the developing T cell compartment broadly in addition to eliciting pathogen-specific T cell responses. Congenital CMV infection causes widespread immune activation and differentiation of the developing T cell compartment [(68, 69), reviewed in (65)]. Moreover, infants exposed to pathogens in utero but not infected (*e.g.*, born to mothers infected with *P. falciparum*, *T. cruzi*, HIV, or hepatitis C virus) have global changes in their T cell immunophenotypes and altered T cell responses to stimulation [(70), reviewed in (71)]. These exposed yet uninfected infants can have reduced T cell responses to vaccines and increased susceptibility to homologous and heterologous infections, perhaps due to inappropriate tolerogenic T cell responses generated in utero (71). Thus, prenatal exposure to pathogens can broadly shape the neonatal T cell compartment with long-term consequences for adaptive immune responses, which should be considered when designing vaccine strategies.

Although capable of generating antigen-specific T cell memory, infant T cell responses are limited compared to adults. Infants have impaired CD4+ T cell responses to *P. vivax* (72), HIV (73), and CMV (74), which may contribute to poor pathogen control. Furthermore, neonates with viral respiratory tract infections (*e.g.*, RSV, influenza, and coronaviruses) preferentially generate T_EM_ rather than tissue-resident memory T cells (T_RM_), which may impair the development of long-lasting memory (75). Given the ongoing outbreak of SARS-CoV-2, a newly emergent coronavirus and ongoing infectious threat to pediatric health, it is important to consider how T cell responses may be age-dependent with distinct implications for disease pathogenesis and vaccine interventions [reviewed in (76)]. These studies suggest that antigen-specific T cell responses are generated in response to certain pathogens, yet effector T cell responses may be favored over memory T cell responses during early life.

#### Early Life T Cell Responses to Vaccines

Infant T cell responses to vaccines have also demonstrated that the T cell compartment is equipped to mount pathogen-specific adaptive responses in early life, but suggests that these are usually diminished compared to adulthood. Infants generate T cell responses following measles vaccination, yet immunization before 6 months of age produce less IFN-γ than adults (77, 78). Moreover, infants receiving the hepatitis B (HepB) vaccine have impaired T cell cytokine production and differentiation compared adults (79). Although the T cell responses to the measles and HepB vaccines are attenuated in infancy compared to adulthood, there is also evidence that infants can mount more adult-like responses to some vaccines.

Infants receiving the Bacillus Calmette–Guérin (BCG) vaccine against *Mycobacterium tuberculosis* have more robust Th1 and pathogen-specific CD8+ T cell responses than adults (80–82). Moreover, priming infants with the BCG vaccine in infancy appears to boost T cell responses to heterologous vaccines, including HepB and oral poliovirus later in life [(83), reviewed in (84)]. *Bordetella pertussis* vaccination also induces a potent Th1 response in young infants and can enhance overall T cell activation (85). Thus, infants can mount vigorous T cell responses following some vaccines despite inherent maturational differences in the early life T cell compartment. Therefore, targeted intervention strategies that account for the distinct nature of the neonatal T cell compartment should be employed to effectively engage T cells in adaptive responses.

## B Cells and Antibody Responses in Early Life

### B Cell Development and the Neonatal B Cell Compartment

#### Development of Antibody Responses and B Cell Receptor (BCR) Diversity in Early Life

Immunoglobulins (Ig) *i.e.*, antibodies, encoded by the B cell receptor (BCR) genes, are the key mediators of adaptive humoral immunity and are composed of different isotypes with distinct functions (*e.g.*, IgM, IgG, IgA, and IgE). While fetal IgM production begins in utero and increases substantially postnatally, endogenous IgG and IgA production, which requires B cell class-switching, remains limited until 6 months of age (86). In order to respond to pathogens, the mammalian adaptive immune system has evolved multiple mechanisms to recognize diverse antigens. BCR combinatorial diversity is achieved when different genes segments in V(D)J loci are recombined to form the V region and junctional diversity is formed when the enzyme terminal deoxynucleotidyl transferase (TdT) adds random N nucleotides at the joints between V(D)J gene segments during DNA recombination. These two sources of diversity can generate, approximately 10^11^ different receptors in the naïve BCR repertoire (87), and more diversity is added when different heavy- and light-chain V regions pair to form the antigen-binding site. Once a BCR recognizes its cognate antigen, somatic hypermutation (SHM) occurs in germinal centers and mutations are introduced to the antibody V regions to improve binding to antigens. Although fetal mature B cells can be detected as early as 9 weeks of gestation, the formation of germinal centers and adaptive humoral responses is greatly attenuated until after birth (10).

By detecting the products of V(D)J DNA recombination generated during B cell development, Rechavi et al. showed that BCR combinatorial diversity begins around 12 weeks of gestation (19). At birth, infants have low levels of SHM, but over time, SHM rates increase. By 2 years of age, SHM rates in non-class-switched IgM and IgD-expressing B cells reach adult levels. However, by 3 years of age, class-switched IgG and IgA-producing B cells only achieve 60 to 75% of adult SHM levels and only reach adult levels at 6 years of age (88, 89). The lower rates of SHM in infants can lead to reduced antibody binding to the antigen. Less differentiated CD27^dull^ memory B cells (which are mostly IgM-expressing) predominate in the infant B cell compartment; the more differentiated CD27^bright^ memory B cells are absent until 3–4 years of age (90). In fact, these CD27^dull^ memory B cells seem to be the progenitor of CD27^bright^ memory B cells, and thus are relatively enriched in early life compared to later in life (90). Functionally, CD27^bright^ memory B cell populations have higher SHM rates, contain more class-switched memory B cells, and generate more potent response to antigens. Differences between adult and infant B cells have also been reported in the Ig complementarity-determining region 3 (CDR3), which is critical for antigen binding. The Ig heavy chain CDR3 (HCDR3) is significantly shorter in fetuses due to fewer N nucleotides at the VDJ junctions compared to term infants, which could be due to lower TdT expression in fetal life (19). HCDR3 maturation is initiated in the third trimester of pregnancy, but HCDR3 lengths do not reach adult levels until 5 months of age (91). Overall, the composition of the memory B cell compartment, limited SHM rates, and shorter HCDR3 lengths during B cell development highlight why infants often have less potent humoral responses to antigens.

#### T Cell-Dependent and T Cell-Independent Humoral Responses to Antigens in Early Life

**Figure 2:**
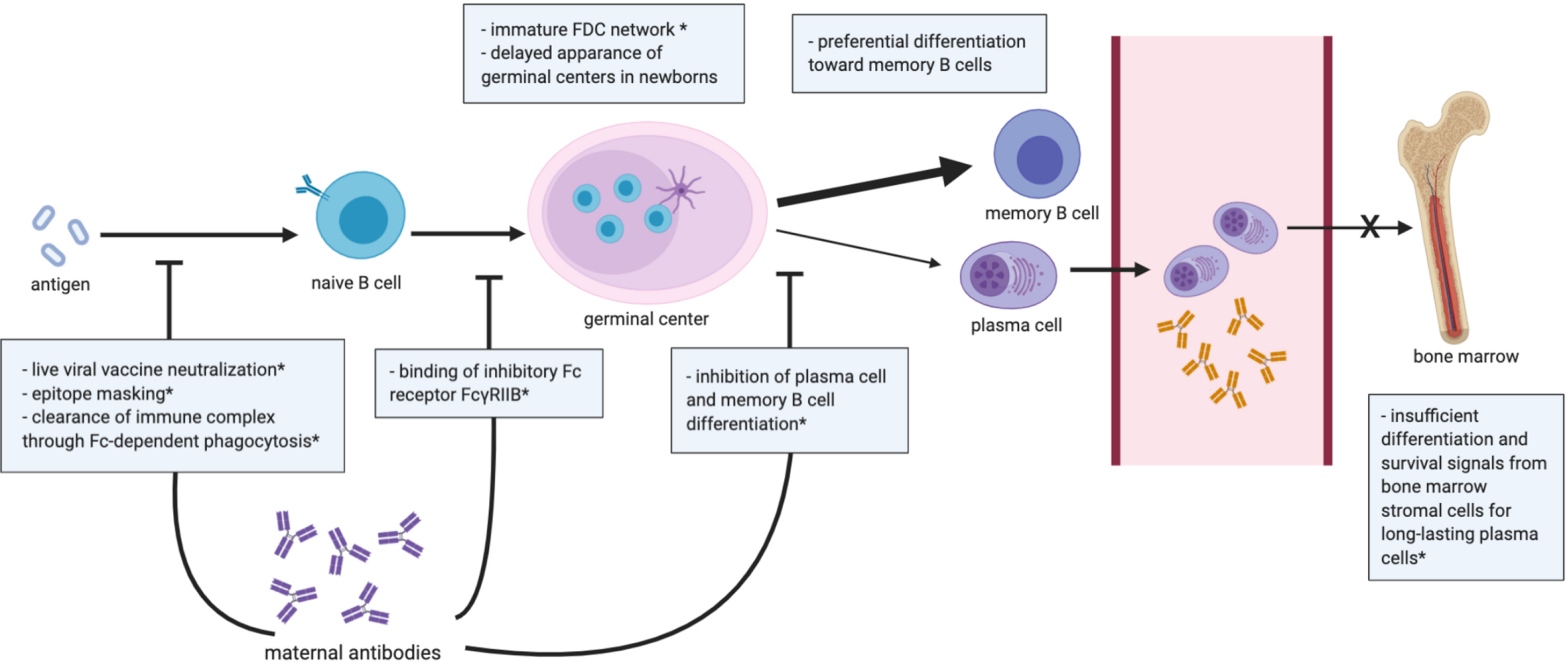
Summary of the B cell compartment and antibody responses in early life. Dampened germinal center reaction is generally observed in infants, and differentiation of activated B cells is skewed toward short-lived memory B cells over plasma cells. Bone marrow in early life is unable to provide an optimal niche for the differentiation of long-lasting plasma cells. Transplacental maternal antibodies influence the neonatal B cell-mediated response by the illustrated mechanisms. *data shown in mice. FDC, follicular dendritic cell.

Overall, early life B cell responses to T cell-dependent (TD) and T cell-independent (TI) antigens are weaker than in adulthood except for a few pathogens and vaccines discussed later (86). As summarized in **Figure 2**, multiple factors contribute to the diminished antibody response in infancy, including cell-intrinsic and cell-extrinsic factors. The gene expression profile of neonatal B cells is distinct from adult B cells, which limits the activation signals neonatal B cells receive from CD4^+^ T cells upon exposure to TD antigens. Specifically, human neonatal B cells express lower levels of the co-stimulatory receptors CD40, CD80, and CD84, resulting in dampened responses to CD40 ligand (CD40L) expressed by T cells (86).

To elicit a potent and durable antibody response, three key structures are involved (**Figure 2**) including: 1) the follicular dendritic cell (FDC) network, 2) germinal centers, and 3) the bone marrow. FDCs capture and retain antigens in immune complexes with complement or antibodies (92). By presenting native antigens to B cells, FDCs play an important role in the nucleation of germinal centers. However, in neonatal mice, FDC precursors fail to differentiate into mature FDCs following B cell-mediated signaling, leading to the absence of effective germinal center responses in neonatal mice until 3 weeks of age (93). Despite no direct evidence in humans, the lack of mature germinal centers in mouse models suggests that immature interactions between FDC and B cells may contribute to the attenuated B cell and humoral responses in early life (94). Another mechanism that may limit early life humoral responses could be the lower expression levels of CD73 in neonatal naïve B cells, as CD73 converts adenosine mono-phosphate into adenosine, an essential immunoregulatory molecule (95). Lower CD73 expression in B cells has also been observed in patients with common variable immunodeficiency, which have limited antibody production, suggesting that decreased CD73 expression in infant B cells may limit humoral responses (96).

Infants generally have lower magnitude and limited persistence of antibody responses following natural infection and vaccination (97, 98), which may result from the inability of early-life bone marrow to establish a pool of long-lived plasma cells. In adults, plasmablasts migrate to the bone marrow after exiting germinal centers, and, with survival signaling from the stromal cells, then differentiate into long-lived plasma cells. However, due to insufficient signals from immature stromal cells, plasmablasts in neonatal mice fail to differentiate into long-lived plasma cells in the bone marrow (98, 99). Furthermore, upon antigen exposure, both human and mouse neonatal B cells show preferential differentiation toward memory B cells instead of long-lived plasma cells (86), possibly due to lower expression of CD40 and CD21 in infant B cells (100, 101).

TI antigens include repetitive, highly valent structures such as polysaccharides, thus B cell responses to TI antigens are key for host defense against encapsulated bacteria. Humans do not develop humoral responses to TI polysaccharides until 1–2 years of age, which is further supported by mouse studies showing that mice (<3 weeks of age) do not respond to immunization with pure pneumococcal polysaccharides (102–104). Due to this impaired TI humoral response, neonates are more susceptible to infections with encapsulated bacteria such as *Haemophilus influenzae* type b (Hib) and *Streptoccoccus pneumoniae*. Neonatal hyporesponsiveness to TI antigens may be due to immaturity of marginal zone B cells and lower numbers of CD27+ memory B cells in early life, as splenic marginal zone B cells are the major subpopulation responding to TI antigens (105). Lower B cell expression of the C3 complement receptor 2 (CR2 or CD21) and decreased levels of C3 complement may further impair humoral responses to TI antigens in early life (105–108). CD21 aids B cell signaling by ligating C3 fragments that opsonize bacterial polysaccharide capsules, so reduced C3 and CD21 levels may render neonatal B cells less sensitive to TI antigens (109). To overcome this limitation of the early life immune system, pediatric vaccines against encapsulated pathogens are conjugated to proteins to recruit T cell help and improve humoral vaccine responses (110–112). Therefore, factors limiting humoral responses to both TD and TI antigens in early life and the nature of the antigen must be considered when designing pediatric vaccines.

#### Unique Factors Influencing Early Life Humoral Responses

Early life antibody responses can be substantially shaped by pathogens, maternal antibodies, and microbial colonization (88, 113–115). The factors most unique to early life that shape humoral responses include maternally transferred antibodies and colonization of the newborn gut microbiota. Due to the limitations to generating antibody responses in early life, neonates rely heavily on passive maternal antibody transfer for protection against infections [reviewed in (116)]. Transplacental IgG transfer, mediated by the neonatal Fc receptor (FcRn), begins during the second trimester and increases throughout gestation (117). Maternal IgG persists in infant circulation for months postnatally, and the importance of maternal antibodies in protecting neonates is underscored by diseases in which transplacental antibody transfer is disrupted. For instance, maternal HIV infection can impair transplacental antibody transfer [reviewed in (118)], which may increase the susceptibility of HIV-exposed yet uninfected infants to heterologous infections (119–122). Passive maternal antibody transfer of mostly secretory IgA (sIgA) and some IgG postnatally through breastmilk also provides protection of the neonate particularly at mucosal surfaces.

Despite the important protection conferred by maternal antibodies, the presence of maternal antibodies can also dampen B cell responses to vaccines in human neonates [reviewed in (123)]. Mechanistic studies on maternal antibody-mediated interference in humans have been lacking, yet these mechanisms have been explored in rodent models [reviewed in (123, 124)]. Proposed mechanisms of interference include: 1) live viral vaccine neutralization, 2) epitope masking, 3) clearance of immune complex through Fc-dependent phagocytosis, 4) binding of inhibitory Fc receptor FcγRIIB, 5) inhibition of the differentiation of plasma cell and memory B cell differentiation (114, 125–127) (**Figure 2**). Maternal antibodies can neutralize live vaccines such as the measles and polio vaccines, which may contribute to the low antibody responses observed in human neonates (124). Maternal antibodies may also inhibit the activation of B cell clones by blocking immunodominant epitopes, which could explain the attenuated neonatal antibody response to vaccines that do not involve live virus (*e.g.*, inactivated and subunit vaccines) (124). Moreover, opsonization by maternal antibodies may facilitate the clearance of vaccine antigen, limiting its availability for infant B cell recognition. Kim et al. also demonstrated in cotton rats that vaccine-specific maternal antibodies may inhibit neonatal B cell activation by crosslinking inhibitory FcγRIIB receptors on B cells (127). Vono et al. demonstrated that high titers of antigen-specific maternal antibodies inhibited the antibody response to autologous immunization in the offspring of vaccinated dams by preventing germinal center B cells from differentiating into plasma and memory B cells, though the formation of germinal centers was not affected (114). Another study of neonatal mice also demonstrated that high but not low or moderate antigen-specific maternal antibody levels hindered pup antibody responses to pneumococcal immunization (128). Thus, maternal antibodies may impact neonatal B cell immunity in many complex ways, perhaps more profoundly than previously appreciated. Understanding the mechanisms regulating maternal antibody transfer and interference with neonatal humoral responses is key for the development of vaccines leveraging passive maternal immunization and for designing pediatric vaccine schedules.

The newborn gut microbiome is primarily colonized shortly after birth, yet a stable, adult-like community is not established until around 3 years of age (129). The microbiome can substantially modulate the host antibody repertoire and responses to infections and vaccines in early life [reviewed in (113)]. Germ-free mice have limited sIgA production, as evidence by smaller Peyer’s patches, lower numbers of CD4^+^ T cells and IgA-producing plasma cells (130), and elevated levels of serum IgE and anaphylaxis (129). Notably, this phenotype can be reversed by introducing commensal microbes before two weeks of age but not after, implying that microbial colonization must be achieved within a narrow perinatal period known as the “window of opportunity” (129–132). However, more studies into how the gut microbiome influences human neonatal humoral responses are needed. Due to differences in microbial colonization, infants and adults can mount distinct antibody response to pathogens. A bias towards HIV envelope (*i.e.*, gp41) specific antibodies that cross react with commensal bacteria has been observed in HIV infected and uninfected adults, but gp41 dominant antibody responses are not observed during pediatric HIV infection (133, 134). This highlights how immune imprinting by the microbiome can modulate antibody specificities and that targeting the microbiome could be a novel strategy for engineering humoral responses to vaccination in early life.

### Antibody Responses to Pathogens and Vaccines in Early Life

#### Early Life Humoral Responses to Pathogens

Despite the attenuated humoral responses in early life to encapsulated bacteria such as Hib and *Streptococcus pneumoniae*, infants and young children are able mount robust antibody responses to some pathogens. For instance, infants generate antibodies that neutralize diverse HIV strains by targeting conserved epitopes (known as broadly neutralizing antibodies, bnAbs). BnAbs develop in a small proportion (10–25%) of HIV-infected adults after years of infection, yet studies have revealed that bnAbs can be detected within 1 year of infection in HIV-infected infants (135, 136). Unlike in HIV-infected adults where plasma neutralization breadth is usually mediated by antibodies against one or two specificities, a high portion of children (63%) develop polyclonal broadly neutralizing antibody responses against HIV (137). Adult bnAbs often have unusual characteristics such as high SHM rates, which may contribute to their delayed development in adults (133). Strikingly, high levels of SHM seem to be less important in pediatric bnAbs as two bnAbs, BF520.1 and AIIMS-P01, isolated from HIV-infected children demonstrate neutralizing potency comparable to adult bnAbs but have much lower SHM rates (6.6 and 7% versus 15.8 to 23.1%) (138, 139). Wiehe et al. compared the number of rare *i.e.*, improbable mutations required for different antibody lineages to acquire heterologous virus neutralization capacity and showed that BF520.1 had fewer improbable mutations than adult bnAbs; however, an improbable mutation, N52A, was essential for neutralization potency, highlighting that such mutation events may be important for generating bnAbs in HIV-infected infants (140). These studies on HIV bnAbs highlight that the developmental pathways for bnAbs in infants may differ from adults and young children may even be better at generating bnAbs (136).

Potently neutralizing antibodies with limited rates of SHM have been observed in RSV infections in early life as well. Goodwin et al. analyzed antibodies isolated from memory B cells of infants (<3 months of age) hospitalized for RSV infection and observed that the majority of the RSV-specific antibodies did not have detectable SHM. Yet, antibodies from each of the infants demonstrated neutralizing activity against RSV and 4 out of 5 had highly potent neutralizing activity (141). Although the authors did not test whether these antibodies prevented RSV reinfection, this study demonstrated that, even with limited levels of SHM, antibodies generated in early life may still have substantial anti-pathogenic *i.e.*, neutralizing capabilities.

#### Humoral Responses to Vaccines in Early Life

Due to immaturity, fewer co-stimulatory receptors, lower SHM rates, limited class-switching, and maternal antibody interference, infant humoral responses to vaccination are often attenuated compared to adulthood. Neonatal immunizations often result in low-titer responses, reduced durability, and poor seroconversion rates. An ideal neonatal vaccine should induce a robust and durable immune response with a single dose at birth, thereby minimizing vulnerability to infections. Yet, only 50% of infants generate a weak neutralizing antibody titer following one dose of the oral polio vaccine, and infants have limited responses to one dose of the diphtheria, pertussis, tetanus (DTaP) and Hib vaccines [reviewed in (124)]. Overall, vaccine-induced antibodies wane to low or undetectable levels 6 to 9 months after the first dose in most infants, Thus, except for the BCG and HepB vaccines, most pediatric vaccines are administered after 2 months of age in a series of booster doses including: the rotavirus vaccine (2 and 4 months), DTaP vaccine (2, 4, 6, and 15–18 months), Hib conjugate vaccine (2, 4, 6, 12–15 months), pneumococcal conjugate vaccine (2, 4, 6, 12–15 months), polio vaccine (2, 4, 6–18 months), seasonal influenza vaccine (>6 months), measles, mumps, and rubella vaccine (12 months), varicella vaccine (12 months), and hepatitis A vaccine (12–18 months) [reviewed in (142)].

Notably, newborns can mount adult-like antibody responses following HepB vaccination (79). This highlights that certain stimuli may be better able to induce a protective immune response *via* vaccination in early life. In fact, when comparing adult and infant responses to HIV vaccines, we observed that children immunized with an oil-in-water emulsion adjuvant (MF59) developed higher antibody titers than adults immunized with the same vaccine, yet no difference was observed between adults and infants when immunized with an Alum-adjuvanted vaccine (143). These results suggest that the choice of the vaccine adjuvant can significantly improve antibody responses early in life, as discussed below.

## Leveraging Knowledge of Early Life Adaptive Immunity

### Vaccination Strategies to Engage Infant Adaptive Immunity

Given the particularities of early life adaptive immunity, vaccination strategies must be specifically tailored to this stage of immune development [reviewed in (144–146)]. As discussed, early life T cell responses are limited due to differences favoring innate and effector over adaptive memory responses, immunotolerance, and an enrichment of RTEs and Tregs. Moreover, the ability to generate long-lived plasma B cells and antibody responses in early life is limited due to immaturity, maternal antibody interference, poor germinal center formation, and reduced T and B cell crosstalk. Work in mouse models suggests that T follicular helper cell (T_FH_) interactions with germinal center B cells are impaired in neonatal immunization [(147–149) reviewed in (145)]; however, studies on early life germinal center responses and T_FH_ cells in humans are lacking [reviewed in (146)].

A promising approach to improve adaptive immune responses to vaccines in early life is through the informed choice of vaccine adjuvants and novel adjuvant combinations, which can stimulate both adaptive immune cells and recruit innate antigen-presenting cells [reviewed in (150)]. For instance, the vaccine adjuvant MF59 elicited robust antigen-specific T cell responses and prevented disease acquisition in infants vaccinated against influenza (151, 152). Studies in neonatal mice suggest that MF59 may improve effecter CD4+ T cell but not T_FH_ responses, though MF59 may enhance germinal center responses compared to Alum (153, 154). Moreover, adjuvants targeting TLRs in early life have been shown to boost early life adaptive immunity in humans and animal models. Adjuvants combining Alum with TLR4 (GLA-squalene emulsion (SE)] or TLR9 (CpG-1826 and IC31) were able to induce T_FH_ responses in neonatal mice (149, 155). Indeed, a TLR7/8 agonist adjuvant 3M-052 overcame the hyporesponsiveness to the pneumococcal conjugate vaccine at birth (156). The TLR4 agonist, monophosphoryl lipid A (MPL), combined with *Quillaja saponaria* (QS-21), an adjuvant called AS01, also enhanced polyfunctional T cell responses in a recent malaria vaccine trial in young infants (157, 158). Furthermore, infant rhesus macaques immunized against HIV with adjuvants such as MF59, AS01, and 3M-052/SE had higher magnitude and avidity antibody responses compared to those immunized with Alum (159). Infant rhesus macaques immunized with the 3M-052/SE TLR activating adjuvant also had higher HIV-specific B cell responses, showing that these adjuvants can elicit strong B cell and humoral responses in early life (159). Moreover, a novel C-type lectin agonist (CAF01) and other pathogen-derived adjuvants (LT-K63 and mmCT) were able to enhance germinal center responses in neonatal mice (154, 155, 160). These studies highlight that newer adjuvants can greatly improve adaptive T cell and humoral immune responses in early life.

Induction of long-lasting vaccine-elicited immunity in early life must also involve optimization of the infant immunization schedule. We have demonstrated in infant rhesus macaques that extending the interval between HIV immunizations from 3 to 6 weeks increased the durability of antibody responses and promoted the generation of high avidity HIV-specific antibodies (159). Increasing the time between immunizations may allow for improved long-lived plasma cell and antigen-specific memory B cell development. However, human studies comparing different immunization intervals for eliciting antigen-specific T cell and humoral responses in early life are lacking, highlighting the need for more research as to the most efficacious interval between booster doses. Moreover, the timing of certain vaccines and coordination of vaccine regimens must be considered given that certain early life immunizations (*e.g.*, BCG and pertussis*)* may also improve subsequent vaccine responses by boosting adaptive immunity broadly.

### Vaccination Strategies Using Passive Maternal Antibody Transfer

Another way to overcome the challenges to generating persistent humoral responses in neonates is to leverage passive maternal antibody transfer. Vaccine-elicited IgG transferred transplacentally and sIgA transferred *via* breast milk can protect neonates while adaptive immunity matures. Maternal vaccination against influenza and pertussis during pregnancy has been shown to be effective in protecting infants (161, 162). Moreover, we recently showed that maternal tetanus, diphtheria and pertussis (Tdap) vaccination during pregnancy improved infant vaccine-specific antibody levels when compared to prenatal vaccination (163). Importantly, additional research into the antibody characteristics, such as subtype and Fc glycosylation, that are most efficiently transferred and that best enhance infant immunity is necessary to maximize the benefits of such interventions [reviewed in (125)].

Considerations of maternal antibody interference with infant B cell development and antibody responses must be considered when integrating maternal vaccination strategies into the pediatric vaccine schedule. A recent randomized control trial of maternal Tdap vaccination suggested that infant vaccination may need to be delayed in the setting of maternal immunization (164, 165). Moreover, maternal immunization in the late secondary trimester led to improved infant antibody levels compared to third trimester vaccination (163). Thus, strategies to improve early life immunity through interventions aimed directly at the neonate and indirectly though maternal immunization must be coordinated.

## Concluding Remarks

Though early life adaptive immune responses to pathogens and vaccinations are limited, the neonatal immune system is clearly capable of generating antigen-specific T and B cell responses. Importantly, the immunologic milieu, immune stimulus, and immune cross-talk from maternal antibodies and microbial colonization are key regulators of infant adaptive immune responses, which must be considered when developing immune-based interventions for early life. In order to harness infant adaptive immunity through vaccination, the informed use adjuvants and optimization of vaccine schedules will be essential. Moreover, immunization strategies targeting mother-infant dyads and passive maternal antibody transfer are a promising strategy for protecting neonates during this vulnerable period of immune development. Significant work remains in this specialized area of human immunology, yet the unique nature of the infant immune system need not be viewed as a barrier to developing effective therapies. By translating our knowledge of early life adaptive immunity, we can develop targeted interventions that improve pediatric health on a global scale.

## Author Contributions

ECS and JLC completed the literature review. ECS, JLC, and RG wrote the first draft of the manuscript. TDB, SRP, and GGF helped conceptualize the manuscript. ECS, JLC, RG, TDB, SRP, and GGF all revised and edited the manuscript. All authors contributed to the article and approved the submitted version.

## Funding

This study was supported by NIH NIAID 2P01AI117915 “Early Life Vaccination to Prevent HIV Acquisition during Adolescence” (SP, GF), NIH NIAID R01AI131978 “Functional Profile to HIV Vaccine Elicited Antibodies in Infants” (GF).

## Conflict of Interest

SP provides consulting services to Moderna, Merck and Co Vaccines, Pfizer Inc. and Sanofi for their preclinical CMV vaccine programs.

The remaining authors declare that the research was conducted in the absence of any commercial or financial relationships that could be construed as a potential conflict of interest.
